# Determinants of The Grade A Embryos in Infertile Women; Zero-Inflated Regression Model

**DOI:** 10.22074/cellj.2017.4214

**Published:** 2017-08-19

**Authors:** Amir Almasi-Hashiani, Azadeh Ghaheri, Reza Omani Samani

**Affiliations:** 1 Department of Epidemiology and Reproductive Health, Reproductive Epidemiology Research Center, Royan Institute for Reproductive Biomedicine, ACECR, Tehran, Iran

**Keywords:** Embryo Research, Assisted Reproductive Technology, Cleavage Stage, Poisson Distribution, Zygote

## Abstract

**Objective:**

In assisted reproductive technology, it is important to choose high quality embryos for embryo transfer. The aim of the present study was to determine the grade A embryo count and factors related to it in infertile women.

**Materials and Methods:**

This historical cohort study included 996 infertile women. The main outcome was the number of grade A embryos. Zero-Inflated Poisson (ZIP) regression and Zero-Inflated Negative Binomial (ZINB) regression were used to model the count data as it contained excessive zeros. Stata software, version 13 (Stata Corp, College Station, TX, USA) was used for all statistical analyses.

**Results:**

After adjusting for potential confounders, results from the ZINB model show that for each unit increase in the number 2 pronuclear (2PN) zygotes, we get an increase of 1.45 times as incidence rate ratio (95% confidence interval (CI): 1.23-1.69, P=0.001) in the expected grade A embryo count number, and for each increase in the cleavage day we get a decrease 0.35 times (95% CI: 0.20-0.61, P=0.001) in expected grade A embryo count.

**Conclusion:**

There is a significant association between both the number of 2PN zygotes and cleavage day with the number of grade A embryos in both ZINB and ZIP regression models. The estimated coefficients are more plausible than values found in earlier studies using less relevant models.

## Introduction

On the basis of The International Committee for Monitoring Assisted Reproductive Technology and the World Health Organization (WHO), infertility is defined as failure to achieve a clinical pregnancy with having regular unprotected intercourse for 12 or more months (1,2). Infertility has been considered as one of the global public health issue in the world by WHO with approximately 80 million infertile couples worldwide (3,4). 

Assisted reproductive technology (ART) is a collection of medical steps for the treatment of infertility. From 1978 (when the first ART baby was born) to 2012, ART has contributed to the birth of more than 5 million infants worldwide (5). In developed countries, approximately 1% of all infants are the product of *in vitro* fertilization (IVF) or intracytoplasmic sperm injection (ICSI) treatments (6). In recent decades, success rates in ART have significantly improved (7). Although ART may help infertile couples to become pregnant, successful outcomes after ART interventions depend on many related factors (5,8), such as embryo quality (8). To make sure that infertile women have a good possibility of getting pregnant it is vital to select and transfer embryos of the best quality; particularly when only one embryo is transferred at a time as higher quality embryos have a better chance of surviving the freezing and thawing process. To the best of our knowledge, few studies have been done to establish the determinants of grade A embryos. The aim of this study was to determine the grade A embryo count and factors related to it in infertile women who presented to Royan Institute, Tehran, Iran. This is the first time such research has been carried out in Iran. 

## Materials and Methods

This historical cohort study consisted of 996 infertile women who presented at the Royan Institute, Tehran, Iran, between January 2012 and December 2013 with primary or secondary infertility. Infertile couples with a grade A embryo count record were included in the study. Couples who did not give consent and cases whose grade A embryo count was missing were excluded from the study. The main independent outcome was the number of grade A (or grade one) embryos. A grade A embryo is the one in which all the blastomeres are the same size and there is no fragmentation within the embryo. Potential confounding factors considered in the analysis were mother’s age in years, body mass index (BMI in kg/m^2^), human chorionic gonadotropin (hCG) injection day, cleavage day (the earliest developmental stage of a fertilized zygote during which there are several mitotic divisions within the zona pellucida), stimulation day, duration of fertility prevention, sperm quality, oocyte quality, duration of infertility, and number of metaphase I and metaphase II oocytes, germinal vesicles (GV), 1 pronucleus (1PN) zygotes, 2PN zygotes, injected oocytes, *in vitro* fertilized oocytes, ampoules and previous ART treatments. 

### Ethics

The study was approved by the Research Ethics Board of the Royan Institute (Ethical code: EC/89/1046). Informed consent was obtained from all participants and they were assured that the results would be published as statistics with no possibility of identifying any personal data. 

### Statistical analysis

The number of grade A embryos is a count variable. Poisson regression is used to model count variables. Zero-Inflated Poisson (ZIP) regression was used to model this count data as it contained excessive zeros. In this study, the outcome variable of interest was the number of grade A embryos, which was zero in 87.18% of cases. The Vuong test was used to confirm the choice of the ZIP model over ordinary Poisson regression. In the Vuong test, a significant z-test indicates that the ZIP model is better than an ordinary Poisson regression model. Also a test for over-dispersion was done using the likelihood ratio test and based on its results; Zero-Inflated Negative Binomial (ZINB) regression and ZIP model was done. Stata software, version 13 (Stata Corp, College Station, TX, USA) was used for all statistical analyses. 

### Results

**Fig.1:**
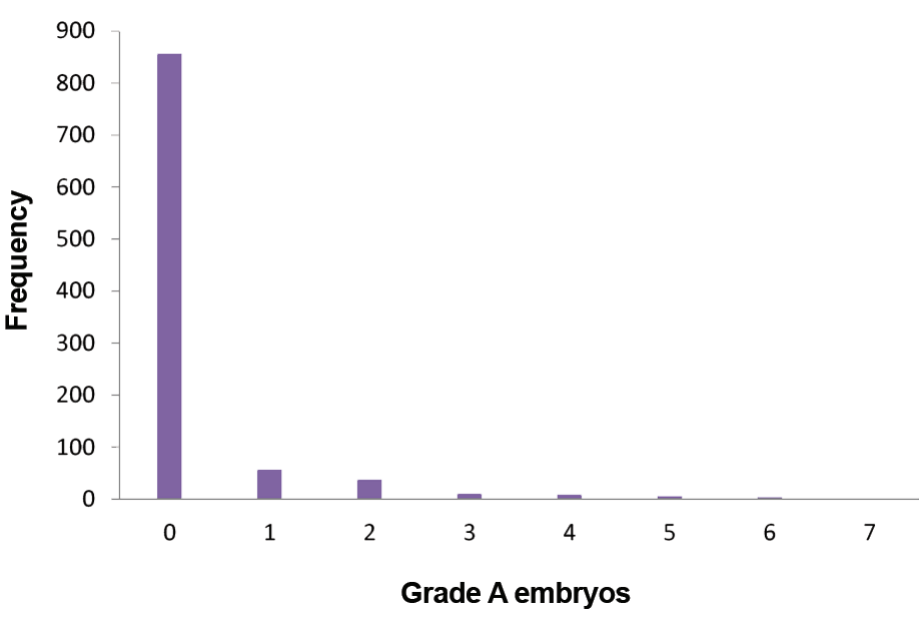
The frequency of grade A embryo in infertile women.

Out of 996 infertile women who were referred to the Royan Institute in Iran for ART, the number of grade A embryos in 857 cases (87.18%) was zero and the mean (SD) number of grade A embryos was 0.27 (0.86), range 0-7 (Fig .1). 

As shown in Table 1, the mean age of the mothers was 35.49 years old and mean BMI was 25.56 kg/m^2^. Other baseline characteristics of the participants are presented in Table 1. The Vuong test showed that there is a significant difference between a ZIP model and an ordinary Poisson regression model (z=4.11, P=0.0001), which indicates that a ZIP regression model is a better fit for this data. On the bases of the crude analysis, cleavage day (P<0.001), 2PN number (P<0.001), number of injected oocyte (P<0.001) and metaphase II number (P<0.001) have a significant relationship with the number of grade A embryos. Previous number of ART treatments (P=0.068) and mother’s age (0.068) approach significance at the P<0.05 level.

In a ZIP regression model which included the potential confounder variables, 2PN number and cleavage day show a significant relationship with the number of grade A embryos, while the association of stimulation day and 1PN number with the number of grade A embryos approached significance. For each increase of 2PN number the expected grade A embryo count increased by 1.32 (95% CI: 1.17-1.48, P=0.001) and for each increase in the cleavage day the expected grade A embryo count decreased by 0.60 (95% CI: 0.39- 0.94, P=0.027). Other variables that were included Table 1: The baseline characteristics of participants in the study Variable Mean SD 95% CI for mean Age of mother (Y) 35.49 5.33 35.16-35.82 BMI (kg/m^2^) 25.56 3.88 25.32-25.81 hCG injection day 12.36 2.55 12.20-12.52 Cleavage day 1.51 0.70 1.46-1.55 Stimulation day 9.70 1.68 9.57-9.84 1PN number 0.22 0.57 0.19-0.26 2PN number 4.33 3.17 4.13-4.53 Number of injected oocyte 7.42 4.15 7.15-7.68 Metaphase I number 0.47 1.12 0.40-0.54 Metaphase II number 7.11 4.22 6.85-7.38 GV number 0.48 1.20 0.40-0.55 Ampoule number 29.20 13.04 28.38-30.02 Previous ART number 1.36 0.48 1.33-1.39 Duration of fertility prevention in year 6.96 5.20 6.63-7.29 Duration of infertility in year 0.89 1.83 0.78-1.01 CI; Confidence interval, BMI; Body mass index, hCG; Human chorionic gonadotropin, PN; Pronucleus zygotes, GV; Germinal vesicles, and ART; Assisted reproductive technology. in the model and displayed in Table 2 have no significant relationships with the number of grade A embryos.

A test for over dispersion using the likelihood ratio test showed that the ZINB model is better than the ZIP model (Chi-square=15.16, P=0.001) and so was performed. The results of ZINB model showed that after adjusting for the potential confounder variables, each unit increase in the cleavage day decreased the expected grade A embryo count by 0.35 (95% CI: 0.20-0.61, P=0.001), and for each unit increase in the 2PN number the expected grade A embryo count increased by 1.45 (95% CI: 1.69- 1.48, P=0.001). Comparison of the two models (e.g. ZIP and ZINB) with Akaike’s information criterion (AIC) indicates that the ZINB model has the smaller AIC (613.5 for ZINB and 626.7 for ZIP).

**Table 1:** The baseline characteristics of participants in the study


Variable	Mean	SD	95% CI for mean

Age of mother (Y)	35.49	5.33	35.16-35.82
BMI (kg/m^2^)	25.56	3.88	25.32-25.81
hCG injection day	12.36	2.55	12.20-12.52
Cleavage day	1.51	0.70	1.46-1.55
Stimulation day	9.70	1.68	9.57-9.84
1PN number	0.22	0.57	0.19-0.26
2PN number	4.33	3.17	4.13-4.53
Number of injected oocyte	7.42	4.15	7.15-7.68
Metaphase I number	0.47	1.12	0.40-0.54
Metaphase II number	7.11	4.22	6.85-7.38
GV number	0.48	1.20	0.40-0.55
Ampoule number	29.20	13.04	28.38-30.02
Previous ART number	1.36	0.48	1.33-1.39
Duration of fertility prevention in year	6.96	5.20	6.63-7.29
Duration of infertility in year	0.89	1.83	0.78-1.01


CI; Confidence interval, BMI; Body mass index, hCG; Human chorionic gonadotropin, PN; Pronucleus zygotes, GV; Germinal vesicles, and ART; Assisted reproductive technology.

**Table 2:** The crude and adjusted incidence rate ratio (IRR) for the number of grade A embryos


	Crude analysis			Adjusted analysis with ZIP model^*^			Adjusted analysis with ZINB model^*^		
	IRR	95% CI	P	IRR	95% CI	P	IRR	95% CI	P

Age of mother (Y)	0.97	0.94-1.01	0.068	0.99	0.95-1.03	0.818	0.97	0.91-1.04	0.545
BMI (kg/m^2^)	0.99	0.95-1.03	0.819	1.01	0.94-1.06	0.878	1.06	0.98-1.15	0.140
hCG injection day	1.03	0.98-1.08	0.181	1.08	0.94-1.23	0.251	1.10	0.92-1.31	0.271
Cleavage day	0.45	0.31-0.65	<0.001	0.60	0.39-0.94	0.027	0.35	0.20-0.61	0.001
Stimulation day	0.99	0.89-1.11	0.962	0.88	0.78-1.01	0.073	0.91	0.75-1.09	0.329
1PN number	1.09	0.90-1.32	0.347	1.52	0.99-2.34	0.054	1.56	0.88-2.75	0.120
2PN number	1.13	1.09-1.17	<0.001	1.32	1.17-1.48	0.001	1.45	1.23-1.69	0.001
Number of injected oocyte	1.08	1.05-1.12	<0.001	0.82	0.63-1.07	0.157	0.78	0.57-1.06	0.123
Metaphase I number	0.98	0.86-1.10	0.750	1.02	0.81-1.28	0.841	1.08	0.82-1.43	0.566
Metaphase II number	1.06	1.03-1.10	<0.001	1.02	0.80-1.30	0.836	1.06	0.79-1.42	0.665
GV number	0.94	0.77-1.15	0.572	1.08	0.86-1.35	0.501	1.09	0.78-1.51	0.607
Ampoule number	0.99	0.97-1.004	0.196	0.98	0.96-1.01	0.363	0.99	0.96-1.02	0.710
Previous ART number	0.90	0.82-1.007	0.068	0.92	0.79-1.06	0.280	0.65	0.35-1.18	0.162
Duration of fertility prevention (Y)	0.99	0.91-1.08	0.964	1.03	0.92-1.15	0.554	.098	0.92-1.05	0.639
Duration of infertility (Y)	0.97	0.94-1.007	0.134	1.01	0.95-1.04	0.999	1.05	0.88-1.25	0.546


CI; Confidence interval, BMI; Body mass index, hCG; Human chorionic gonadotropin, PN; Pronucleus zygotes, GV; Germinal vesicles, ART; Assisted reproductive technology, ZIP; Zero Inflated Poisson, ZINB; Zero Inflated Negative Binomial, and *; Adjusted for other variables in the Table.

## Discussion

Despite the remarkable advances in our understanding of embryo development, identifying embryos with higher chance of survival and development is still challenging. Prior studies have noted that good morphology grade embryos are important in ART due to their association with significant improvement of implantation and live birth rates (9). This study set out with the aim of assessing the factors affecting the number of grade A embryos retrieved from infertile women. ZIP and ZINB regression were used to model number of grade A embryos, as a count variable with an excess of zero counts.

The results obtained from the likelihood ratio test showed that the estimated mean and variance are substantially different; therefore a ZINB regression model is preferred to a ZIP model. Although both the ZIP and ZINB model fitted the data well, and the same variables were selected by both models, the AIC showed that ZINB is a better model to determine the factors associated with the number of grade A embryos, since a model with a lower AIC is more plausible than one with a higher AIC (10). The estimated coefficients obtained in this study are more plausible than those found in earlier studies using less relevant models.

Strong evidence of a relationship between cleavage day and number of grade A embryos was detected in the crude analysis, but it was not due to confounding by other variables since earlier cleavage was still associated with a greater number of grade A embryos after being adjusted for other variables in ZINB model as well as the ZIP model. This finding is in compliance with previous studies which identified the role of cleavage stage as a key role for successful ART cycles (11-14). It can be speculated that embryos with earlier cleavage stem from oocytes with better synchronized cytoplasmic and nuclear maturation and/or a higher metabolic fitness, i.e. the competence and availability of, for example mRNA, mitochondria, etc. (15). It is also demonstrated that cells with aneuploidy chromosomal status cleave more slowly in general and early cleavage is an indicator of the chromosomal status of the embryo as well (16). This result may be clinically useful in terms of considering early cleavage as a criterion when a number of embryos have similar characteristics at time of transfer (15).

Another important finding in the adjusted analysis, revealed by both the ZINB and ZIP models was the positive association between the number of embryos originated from 2PN zygotes and the number of grade A embryos. This finding is consistent with those of previous studies showing that 2PN zygotes tend to cleave to embryos with sufficient morphological quality (12, 17, 18). It is likely that the association between the number of embryos originated from 2PN zygotes and number of grade A embryos occurred because of the significant relationship between cleavage day and number of grade A embryos. This explanation seems plausible since previous findings show that embryos with better a pronuclear pattern cleave earlier and faster and can result in better quality embryos (19, 20).

Age was marginally significant in the crude analysis, but it was not significantly associated with the number of grade A embryos after adjusting for other confounding variables in either the ZINB or the ZIP multivariate models. Although this result differs from the results of some published studies (21, 22), it is consistent with that of the Penzani et al. (23). The number of injected oocytes, number of mature (metaphase II) oocytes and number of previous ART cycles, which are potentially effective factors in pregnancy (24, 25), were significant in the crude analysis but not in the adjusted analysis in either the ZINB or ZIP models. Unlike previous studies (21), the present study did not show any association between maternal BMI and number of grade A embryos. In the current study, some factors related to embryo quality have not been examined. For example, further studies need to be conducted to analyze the effect of variables related to maternal nutritional status and parental karyotype.

## Conclusion

The number of 2PN zygotes and cleavage day have a significant relationship with the number of grade A embryos in both ZINB and ZIP regression models. 
